# Risk factors of functional disability among community-dwelling elderly people by household in Japan: a prospective cohort study

**DOI:** 10.1186/1471-2318-14-93

**Published:** 2014-08-26

**Authors:** Emiko Saito, Shouzoh Ueki, Nobufumi Yasuda, Sachiko Yamazaki, Seiji Yasumura

**Affiliations:** 1 Department of Nursing Sciences, Graduate School of Human Health Sciences, Tokyo Metropolitan University, 7-2-10, Higashiogu, Arakawa, Tokyo, 116-8551, Japan; 2 Department of Medical Science & Welfare, Tohoku Bunka Gakuen University, 6-45-1 Kunimi, Aoba, Sendai 981-8551 Miyagi, Japan; 3 Department of Public Health, Kochi Medical School, Kohasu, Okoh-cho, Nankoku, Kochi 783-8505, Japan; 4 Department of Psychology, Faculty of Human Studies, Bunkyo Gakuin University, 1196 Kamekubo, Fujimino, Saitama, 356-8533, Japan; 5 Department of Public Health, Fukushima Medical University, 1 Hikariga-oka, Fukushima 960-1295, Japan

**Keywords:** Elderly, Functional disability, Households, Long-term care

## Abstract

**Background:**

Although the number of elderly people needing care is increasing rapidly in the home setting in Japan, family size and ability to provide such support are declining. The purpose of this study was to identify the risk factors of functional disability by household composition among community-dwelling elderly people.

**Methods:**

A total of 1347 elderly people aged 70 years and over participated in a baseline geriatric health examination for this prospective cohort study. In the health examination, we conducted an interview survey using a questionnaire in July 2004 and July 2005. Questionnaire items covered the following: age, sex, household, medical history, instrumental activities of daily living, intellectual activity, social role, Motor Fitness Scale, falls experienced during the past year, Dietary Variety Score, frequency of going outdoors, cognitive impairment, and depressive status. We defined the occurrence of functional disability as certification for long-term care needs of the subjects. The certification process started with a home visit for an initial assessment to evaluate nursing care needs using a questionnaire on current physical and mental status. The onset of functional disability was followed from July 2004 to March 2011. Cox proportional hazard regression analysis was used to estimate the risk factors related to the onset of functional disability, adjusted for age and sex.

**Results:**

Of the 1084 participants, 433 were male (39.9%), and the average age was 77.8 (standard deviation, 5.4). Up to March 2011, functional disabilities occurred in 226 participants (20.9%). Elderly people living only with their children demonstrated a significantly higher risk for functional disability than the three-generation household group (hazard ratio, 1.61; 95% confidence interval, 1.08–2.40). The risk factors for functional disability varied according to household group.

**Conclusions:**

In Japan, the number of vulnerable households with elderly people in need of care has increased steadily over the years. Appropriately identifying the risks related to functional disability requires a means of assessment that takes the household composition into consideration.

## Background

Households in Japan, including those with elderly people, have become increasingly characterized by diminishing size and reduced care functions. The proportion of the population in the 65 or older age-group amounted to 24.1% in 2012, with the ratio of the elderly to the working-age population (aged 15–64 years) having been 1:2.6 [1]. Because of the effects of social change and urbanization, families are also becoming more nuclear: the average number of people per household diminished to 2.57 in 2012 [2]. The number of three-generation households with an elderly person aged 65 or older has decreased (3.35 million households, 16.2%, as of 2010; 4.14 million households, 26.5%, as of 2000; 4.27 million households, 39.5%, as of 1990), and the number of spouse-only households has increased (6.19 million households, 29.9%, as of 2010; 4.23 million households, 27.1%, as of 2000; 2.31 million households, 21.4%, as of 1990). Single-person household have also increased in number (5.02 million households, 24.2%, as of 2010; 3.08 million households, 19.7%, as of 2000; 1.61 million households, 14.9%, as of 1990) [1]. The number of three-generation households with an elderly person aged 65 or older has decreased: 3.20 million households (15.3%) as of 2012. Likewise, the number of spouse-only households has increased: 6.33 million households (30.3%) as of 2012. The number of single-person household has also increased: 4.87 million households (23.3%) as of 2012 [2]. This trend is expected to continue, and the number of spouse-only and single-person households is estimated to increase to approximately 6.33 and 7.30 million in 2030, respectively [3]. It has been predicted that the proportion of couple-only and single-person households will account for approximately 70% of all households with an elderly person aged 65 or older [1]. Despite the decline in family resources and functions, the number of elderly people with functional disability is on the rise (2.88 million as of 2001; 4.91 million as of 2010) [1]. The risk factors related to the functional decline of elderly people in different households are still not fully clear.

Several cross-sectional studies have investigated household factors related to mortality, risk of disease, admission to nursing homes or hospitals, self-related health, depressive status, and poverty risk among elderly people [4-9]; however, there is a lack of longitudinal data relating to vulnerable households. In many developed countries, the household composition has shifted toward smaller size and more diverse range, and that particularly applies to Japan’s aging society. The development toward smaller household sizes and reduced family functions can potentially cause difficulties for elderly people with functional disability in a home-care setting. For example, the number of elderly people living alone and those living only with a spouse has significantly increased, and several risks have been identified for this age-group [5,10,11]. Household composition is an important factor when assessing needs and providing health-care services for elderly people, and it includes the physical, mental, and social functional conditions of such people as well as their caregivers [6,12]. However, few reports have examined the relationship between these diverse household factors related to the elderly and their functional disability.

Functional disability in community-dwelling elderly people is a frequent cause of admission to a hospital or nursing home and the use of long-term care services.[13,14]. Japan’s long-term care insurance (LTCI) system, which includes services to prevent functional disability among the elderly, started in 2000. The number of elderly people requiring care under the LTCI system has increased every year, and it was 5.06 million in 2010. Despite the rise in the number of elderly people requiring care, it is becoming more difficult for family members to provide care for elderly relatives. The composition of households with elderly people is characterized by ever-smaller size. Brown et al. [15] reported that after recovery from an illness, elderly people living alone may need to move away from the local community where they have lived for a long time. Elderly people living alone find it harder to obtain social support in an environment with unknown neighbors than those living with others [16]. Furthermore, it has been reported that elderly males living alone have a higher mortality than those living with others [17]. From the perspective of mental health, fewer people living in a household has been associated with loneliness among the elderly [18]. However, few reports have dealt with the actual conditions for elderly people living only with their spouse or living alone.

Because the number of elderly people needing care and that of smaller households with such individuals are predicted to increase, it is important to determine the risk factors for households over the next decade. However, few studies have focused on household composition for elderly people. Accordingly, the purpose of the present study was to identify the risk factors of functional disability among community-dwelling elderly people by household composition in a region of Japan.

## Methods

### Subjects and setting

In July 2004 and July 2005, we conducted a baseline survey during geriatric health examinations held at a community health center by means of a self-rated questionnaire [19]. The participants for the baseline survey were selected from 1523 elderly people aged 70 years or older residing in a region within Fukushima Prefecture, Japan. Of these, 176 were excluded from the baseline survey owing to functional disability and hospitalization, which left 1347 participants. We aimed to survey all the community-dwelling elderly individuals aged 70 years and older; we divided the community into two areas and conducted each survey in 2004 and 2005. For the elderly people who did not undergo this geriatric health examination at the community health center, we visited their homes and carried out health examinations and interviews. The health examinations and home visits were conducted by public health nurses, nutritionists, home caregivers, and physicians, who were briefed in advance. They had experience in health examinations and home visits for elderly people.

New occurrences of functional disabilities were recorded by the health-care professionals up to 31 March 2011. In the observation period, the participants were monitored in terms of the onset of functional disability by means of the LTCI database. The longest observation period was 2463 days, and the average was 2316.3 days.

To make use of the long-term care service provided by LTCI, care-needs certification has to be recognized by the long-term care-needs certification board. Consisting of physicians, nurses, and other experts in health and social welfare services, the board determines whether the initial computer-based assessment is appropriate; it considers the applicant’s primary-care physician’s statement and also the notes written by the assessor during a home visit [20]. There are seven levels of criteria for LTCI certification: requiring support level 1 and level 2; requiring long-term care from level 1 to level 5. LTCI certification excludes non-eligible applicants. “Requiring support level” signifies care on an ongoing basis to reduce or prevent a condition that limits, partially or wholly, the basic movements for daily activities owing to physical or mental problems. “Requiring long-term care level” means that care is needed on an ongoing basis for all or some basic movements in daily activities because of physical or mental problems. When a participant first applied for long-term care (LTC) services and was certified as being in one of the seven LTC need levels (ranging from requiring support level 1 or 2 to requiring LTC level 5), we regarded this as a functional disability occurrence. The certification date was set as the application date because the certified level of care needed is effective retroactively to the application date for LTC services.

The participants received a document in advance from the local government that explained the geriatric health examinations, and it explained the purpose of our survey at the time of the health examination or home visit. Information about functional disabilities was coded using numbers to prevent identification, and a data file stripped of personal identification information was employed for our analysis. All participants provided their informed consent. This study was implemented as part of the local government’s health-service projects, and we received approval from the Ethics Committee Review Board of Fukushima Medical University (No. 1061). To determine the participant outcome at the end of the observation period, we collected information about functional disability status and change of status, such as death or relocation, from the list of beneficiaries of care-needs certification reviews and the list of people with changes in LTCI eligibility.

### Measurements

#### Outcome variable

For functional disability, we collected data at the onset of certification for LTC from recipients eligible for LTCI [20]. The certification process started with a home visit for an initial assessment to evaluate nursing care needs using a questionnaire on current physical and mental status. The results were assigned a care-needs level, and the Nursing Care Needs Certification Board determined whether the initial assessment was appropriate. The observation period ended on 31 March 2011. We regarded the certification occurrence to be when a participant first applied for LTC services.

#### Independent variables

The items included in the baseline survey were age, gender, household, self-reported medical history (stroke, hypertension, heart disease, osteoporosis, diabetes mellitus), the Tokyo Metropolitan Institute of Gerontology index of competence (TMIG) [21], the Japanese version of the Motor Fitness Scale (MFS) [22], history of falling in the past year [23], the Dietary Variety Score (DVS) [24], the frequency of going outdoors [25], cognitive impairment, and the shorter version of the Geriatric Depression Scale (GDS). The TMIG consists of a 13-item index, and it comprises three sublevels of competence: (i) instrumental activities of daily living (IADL) (five items: the ability to use public transport, buy daily necessities, prepare a meal, pay bills, and handle banking matters—rated on a yes/no basis); (ii) intellectual activity (IA) (four items: the ability to complete forms, read newspapers, read books or magazines, and take interest in television programs or news articles on health-related matters—rated on a yes/no basis); and (iii) social role (SR) (four items: the ability to visit friends, give advice to confiding relatives and friends, visit someone at hospital, and initiate conversation with younger people). The response to each item was designed as yes (able to do) or no (unable to do), and it was scored as 1 for yes and 0 for no. A full score for the 13 items was 13 points. A higher score indicates a higher competence of functional status for older adults. The reliability and validity of the TMIG index of competence have been tested [21].

The MFS is a self-rated measurement scale that comprises 14 items in three subscales (mobility, six items; strength, four items; and balance, four items) to assess the physical performance of older adults [22]. The total score is 14, with higher scores representing better physical performance. The reliability and validity of the MFS have been tested [22]. The DVS [24] was obtained based on the intake frequency of 10 major food groups: meat, fish and shellfish, eggs, milk, soybean products, potatoes, green and yellow vegetables, fruits, seaweed, and fat and oil. The total score was determined by defining “eat almost every day” as 1 point and “eat once every two days”, “eat once or twice a week”, or “seldom eat” as 0 points. Those who scored at least 9 points were considered to have a well-balanced diet. The degree of housebound status was measured by frequency of going outdoors: more than once a week; one to three times a month; and seldom. Cognitive function was evaluated in terms of whether or not the participant had difficulty functioning in daily life through cognitive dysfunction. This was likewise assessed in terms of three self-reported stages: no cognitive dysfunction or no inconvenience in daily life; slight inconvenience in daily life; and serious inconvenience in daily life. The GDS [26-28] is a 15-item self-reported assessment: a score of 5 or higher of a possible 15 points indicates depression.

### Statistical analysis

Of the 1347 elderly people who were scheduled to undergo health examinations, 1290 (95.8%) responded to the survey and answered the questions about their household composition. We excluded 57 elderly people who were unable to be interviewed, either because they refused or were absent during a home visit. Among the 1290 participants, we analyzed 1084 people who we were able to classify into four types of household composition: living in a three-generation household; living only with their children; living only with their spouse; and living alone. Of the 1290 elderly people, 206 participants were classified as having a household composition that was not one of the above four types.

The onset of functional disability for a participant was defined when their support or care needs were newly certified for LTCI during the observation period. To examine the relationships excluding the effects of age at the time of the health examinations and sex, we performed a Cox regression analysis with an onset of functional disability as the endpoint, age and sex as covariates, and the 136 people who died before applying for LTCI and the 12 people who relocated during the observation period as the censored cases. The proportional hazards assumption for the model was checked by examining log-minus-log transformed Kaplan-Meier estimates of the survival functions for the functional disability groups. An alpha level of 0.05 was employed for all statistical tests. We used SAS version 9.3 for Windows (SAS Japan Inc., Tokyo) for analysis.

## Results

Of the 1084 participants, 433 were male (39.9%), and the average age was 77.8 (standard deviation, 5.4); 226 participants (20.9%) had a new occurrence of functional disability. Among the participants with functional disability, 42.5% (96/226) were assigned a low level of need for long-term care (support level 1, support level 2, or care level 1) and 57.5% (130/226) were assigned higher levels. The 1084 participants were classified into four groups according to household composition: three-generation households (798 participants, 73.6%); those living only with their children (101 participants, 9.3%); those living only with their spouses (131 participants, 12.1%); and those living alone (54 participants, 5.0%) (Table 1). As an index of internal consistency, the Cronbach alpha for the TMIG index and MFS was 0.830 and 0.879, respectively.

**Table 1:** Characteristics of the elderly people according to household composition at the baseline (n = 1084)

**Parameters**	**Three-generation (n = 798)**	**Children-only (n = 101)**	**Spouse-only (n = 131)**	**Living alone (n = 54)**	***p*** **value**
	**n (%) or mean (SD), SD: standard deviation**				
Average age	77.9 (SD 5.4)	79.6 (SD 5.6)	76.1 (SD 4.5)	78.8 (SD 5.8)	<0.001
Age-groups					
70–74	241 (30.2)	21 (20.8)	54 (41.2)	16 (29.6)	0.001
75–79	279 (35.0)	32 (31.7)	51 (38.9)	15 (27.8)	
80–84	190 (23.8)	28 (27.7)	18 (13.7)	12 (22.2)	
> = 85	88 (11.0)	20 (19.8)	8 (6.1)	11 (20.4)	
Gender, men	325 (40.7)	26 (25.7)	66 (50.4)	16 (29.6)	0.001
History of disease					
Hypertension	378 (47.4)	54 (53.5)	54 (41.2)	25 (46.3)	0.320
Heart disease	88 (11.1)	16 (15.8)	11 (8.4)	3 (5.6)	0.173
Osteoporosis	86 (10.8)	14 (13.9)	12 (9.2)	9 (16.7)	0.395
DM	85 (10.7)	6 (6.0)	11 (8.4)	4 (7.4)	0.402
Stroke	60 (7.5)	10 (9.9)	8 (6.1)	1 (1.9)	0.294
TMGI					
IADL (0-5)	4.5 (SD 1.0)	4.3 (SD 1.3)	4.7 (SD 0.9)	4.7 (SD 0.7)	0.034
IA (0-4)	3.3 (SD 1.0)	2.9 (SD 1.4)	3.5 (SD 1.0)	3.0 (SD 1.2)	<0.001
SR (0-4)	3.5 (SD 0.9.)	3.1 (SD 1.1)	3.6 (SD 0.9)	3.1 (SD 1.1)	<0.001
MFS	9.6 (SD 3.9.)	8.9 (SD 4.0)	10.8 (SD 3.5)	9.2 (SD 3.9)	0.001
Falls experienced during the past year					
Presence	210 (26.3)	25 (24.8)	29 (22.1)	18 (33.3)	0.449
DVS^1)^	4.8 (SD 2.4)	4.8 (SD 2.6)	5.5 (SD 2.3)	3.8 (SD 2.1)	<.0001
DVS^1)^ < =8	727 (91.1)	88 (88.9)	114 (87.0)	53 (98.1)	0.059
Frequency of going outdoors					
< 1/week	159 (19.9)	33 (32.7)	18 (13.7)	18 (33.3)	<0.001
Cognitive function					
Mild or severe	41 (5.1)	7 (6.9)	6 (4.6)	4 (7.4)	0.759
GDS^2)^ > =5	267 (33.5)	39 (40.6)	37 (28.2)	22 (40.7)	0.286

^1)^Missing data for 16. ^2)^Missing data for 48. Student’s *t* test, *χ*^2^ test, or Fisher’s exact test. *TMIG*, Tokyo Metropolitan Institute of Gerontology index of competence; *IADL*, Instrumental Activities of Daily Living; *IA*, Intellectual activity; *SR*, Social role; *MFS*, Motor Fitness Scale; *DVS*, Dietary Variety Score; *GDS*, Geriatric Depression Scale.

The following eight items showed significant differences among the four groups: average age and age-group, gender, IADL, IA, SR, MFS, DVS, and frequency of going outdoors. Using the Bonferroni correction, the average age of the elderly living in spouse-only households was found to be significantly lower than that for the other three groups: three-generation households (*p* = 0.002); children-only households (*p* < 0.001); and living-alone households (*p* = 0.010). The average age of the children-only households was found to be significantly higher than for the three-generation households (*p* = 0.010).

Table 2 presents the rate of functional disability for each of the household groups and the hazard risk for each group compared with the three-generation household group adjusted for age and sex as covariates. The children-only group demonstrated a significantly higher risk for functional disability than the three-generation household group: hazard risk (HR), 1.61; 95% confidence interval (CI), 1.08–2.40.

**Table 2:** Comparison between the three-generation households and other households with hazard risks for functional disability

**Household**	**Number**	**Onset of functional disability**	**HR**	**95% CI**	***p*** **value**
Three-generation	798	163			
Children-only	101	29	1.61	1.08-2.40	0.020
Spouse-only	131	19	0.91	0.56-1.48	0.693
Living alone	54	15	1.13	0.86-1.48	0.377

*HR*, Hazard risk; 95% *CI*, 95% confidence interval.

Cox regression analysis. All variables were adjusted for covariates of age and sex.

We performed a Cox regression analysis with a new occurrence of functional disability as the endpoint, age and gender as covariates, and 14 variables for each of the four groups (Table 3). In the three-generation household group, nine variables were significantly associated with functional disability: history of DM (HR = 2.16; 95% CI, 1.45–3.22); history of stroke (HR = 2.22; 95% CI, 1.39–3.55); IADL (HR = 0.77; 95% CI, 0.69–0.85); IA (HR = 0.82; 95% CI, 0.72–0.94); SR (HR = 0.74; 95% CI, 0.64–0.86); MFS (HR = 0.90; 95% CI, 0.86–0.94); falls experienced during the past year (HR = 1.41; 95%CI, 1.02–1.95); frequency of going outdoors (HR = 2.46; 95% CI, 1.76–3.44); and depressive status (HR = 1.71; 95% CI, 1.25–2.35). In the spouse-only group, nine variables were significantly associated with functional disability: history of stroke (HR = 4.57; 95% CI, 1.26–16.61); IADL (HR = 0.52; 95% CI, 0.38–0.71); IA (HR = 0.52; 95% CI, 0.36–0.75); SR (HR = 0.56; 95% CI, 0.37–0.85); MFS (HR = 0.78; 95% CI, 0.69–0.88); falls experienced during the past year (HR = 4.70; 95% CI, 1.71–12.91); DVS (HR = 5.30; 95% CI, 1.34–20.94); frequency of going outdoors (HR = 3.95; 95% CI, 1.27–12.29); and depressive status (HR = 4.59; 95% CI, 1.68–12.57). In the living-alone group, two variables were significantly associated with functional disability: heart disease (HR = 5.67; 95% CI, 1.06–30.14) and depressive status (HR = 5.19; 95% CI, 1.33–20.31).

**Table 3:** Adjusted hazard ratios (HRs) by household composition at follow-up: bivariate analysis

**Parameters**	**Three-generation (n = 798)**	***p*** **value**	**Children-only (n = 101)**	***p*** **value**	**Spouse-only (n = 131)**	***p*** **value**	**Living alone (n = 54)**	***p*** **value**
History of disease								
Hypertension	1.11 (0.81–1.53)	0.510	0.47 (0.22–1.03)	0.059	1.06 (0.40–2.82)	0.912	0.92 (0.33–2.58)	0.881
Heart disease	1.51 (0.98–2.33)	0.065	0.79 (0.23–2.70)	0.700	0.12 (0.01–1.16)	0.067	5.67 (1.06–30.14)	0.042
Osteoporosis	0.92 (0.57–1.50)	0.748	0.49 (0.14–1.74)	0.269	0.44 (0.06–3.34)	0.425	0.51 (0.11–2.28)	0.377
DM	2.16 (1.45–3.22)	<0.001	2.31 (0.67–7.94)	0.183	3.26 (0.92–11.59)	0.068	1.18 (0.15–9.51)	0.879
Stroke	2.22 (1.39–3.55)	0.001	1.16 (0.34–3.94)	0.817	4.57 (1.26–16.61)	0.021	-	-
TMIG								
IADL	0.77 (0.69–0.85)	<0.001	0.99 (0.71–1.39)	0.955	0.52 (0.38–0.71)	<0.001	0.66 (0.39–1.11)	0.119
IA	0.82 (0.72–0.94)	0.004	0.83 (0.65–1.06)	0.132	0.52 (0.36–0.75)	<0.001	1.25 (0.78–1.99)	0.358
SR	0.74 (0.64–0.86)	<0.001	0.81 (0.58–1.14)	0.227	0.56 (0.37–0.85)	0.007	0.95 (0.60–1.52)	0.836
MFS	0.90 (0.86–0.94)	<0.001	0.94 (0.85–1.05)	0.278	0.78 (0.69–0.88)	<0.001	0.97 (0.84–1.11)	0.615
Falls experienced during the past year								
Presence	1.41 (1.02–1.95)	0.040	0.53 (0.18–1.52)	0.237	4.70 (1.71–12.91)	0.003	2.63 (0.87–7.94)	0.087
DVS < =8	1.05 (0.53–2.06)	0.894	0.98 (0.35–2.77)	0.967	5.30 (1.34–20.94)	0.017	-	-
Frequency of going outdoors								
< 1/week	2.46 (1.76–3.44)	<0.001	1.99 (0.91–4.37)	0.086	3.95 (1.27–12.29)	0.018	1.26 (0.40–3.99)	0.700
Cognitive function								
Mild or severe	1.60 (0.93–2.75)	0.093	1.64 (0.49–5.50)	0.423	2.23 (0.49–10.19)	0.300	-	-
GDS > =5	1.71 (1.25–2.35)	0.001	1.02 (0.45–2.33)	0.960	4.59 (1.68–12.57)	0.003	5.19 (1.33–20.31)	0.018

Cox regression analysis. All variables were adjusted for covariates of age and sex.

Data are given as hazard ratios (95% confidence interval). *TMIG*, Tokyo Metropolitan Institute of Gerontology index of competence; *IADL*, Instrumental Activities of Daily Living; *IA*, Intellectual activity; *SR*, Social role; *MFS*, Motor Fitness Scale; *DVS*, Dietary Variety Score; *GDS*, Geriatric Depression Scale.

## Discussion

This study found that the children-only household group demonstrated a higher risk for functional disability than the three-generation household group, and the factors associated with functional disability differed depending on household composition. In this regard, the risk in children-only households has not been reported. Life-course analysis has shown that critical periods exist, for example those related to family life events or family life course, with respect to health disparities [29]. Among families in East Asian countries, including Japan, the number of households with elderly parents and unmarried children has increased. Though elderly people living alone face many social problems, such as obtaining nursing care, isolation, and low income, few studies have investigated this issue. The present study examined the vulnerability of such households. The poor family functions of the children-only households were probably caused by vulnerability during the family life course.

The factors associated with functional disability were as follows. In the three-generation household group, history of DM, history of stroke, IADL, IA, SR, MFS, falls experienced during the past year, frequency of going outdoors, and GDS were associated with functional disability. In the children-only household group, history of hypertension was linked with functional disability. In the spouse-only household group, history of stroke, IADL, IA, SR, MFS, falls experienced during the past year, DVS, frequency of going outdoors, and GDS were associated with functional disability. In the living-alone household group, history of heart disease and GDS were linked with functional disability. Functional disability has also been reported to be associated with experiencing a fall, depression, IADL, frequency of going outdoors, and MFS [10,30,31]. In the present study, variables similar to those in other studies were found for each of the household groups, although they differed by group. However, no items related to cognitive function demonstrated a significant association with functional disability among any of the household groups. We used a self-reported questionnaire for the item of cognitive function. The subjective preference of respondents may have affected this result. Though the rate of functional disability was greatest among children-only households, all used independent variables were not statistically significant with functional disability. One reason why is that advancing age itself might be a strong risk factor than other risk factors observed in other group. So, we suppose no significant risk factors could not be detected in children-only households.

In the present study, the proportion of newly occurring functional disability among elderly people aged 70 years or over was 20.9%. In previous studies involving participants aged 65 or older, the reported occurrence rates were 4.5% for 24 months [10], 8.0% for 40 months [30], and 8.6% for 36 months [31]. A possible reason for the higher incidence in the present study is that our participants were older. Because most local regions of Japan have a high population-aging rate, there is the possibility that the occurrence of functional disability is likewise high.

We found depressive status to be associated with functional disability in the three-generation, spouse-only, and living-alone household groups. Depressive status has been reported to be associated with functional disability [32]. Furthermore, Russell and Taylor [33] reported that living alone is associated with higher levels of depressive symptoms. When nurses provide vulnerable families with services, it is necessary to consider mental health care in the community setting. Previous studies have reported that the factors associated with functional disability among elderly people living alone include age, not participating in social activities, talking to a friend over the phone less than once a week [34], and lack of social support [35]. In one study comparing three household groups (living alone, spouse only, and living with a child), it was found that for elderly people living alone, participating in social activities and having close contact with neighbors or friends were related to quality of life [36]. Our study did not find significant associations for intellectual activity or social role in the living-alone household group. However, those factors were associated with functional disability in the three-generation and spouse-only household groups. It is possible that the definitions of social activity used in previous studies differ from those employed in the present one. This is a significant factor when interpreting the results of this study.

We found the rate of functional disability to be greatest in the children-only households and that it progressively decreased in the living-alone, three-generation, and spouse-only households. In a cross-sectional analysis, Wang et al. [37] found that living arrangements were significantly associated with activities of daily living. They also suggested that unmarried elderly people living only with their children had significantly greater disability than those in other living arrangements. Murayama et al. [6] reported that functional and cognitive conditions were associated with household composition, especially among elderly people living alone. The present study did not analyze in detail the family members’ characteristics and living conditions for elderly subjects who lived with family. In Japan, the number of three-generation households is decreasing, though the numbers of single-person households, spouse-only households, and households consisting of parents and their unmarried children are increasing; household compositions are also becoming more diverse. The incidence of elder abuse is highest in households consisting of a parent or parents and their unmarried children [2]. To assess the health status and living conditions of elderly people toward preventing functional disability, it is necessary to identify more diversified household compositions and the risk factors associated with each composition.

Some limitations of this study are as follows. First, because the four household groups had different numbers of the onset of functional disability, this study may have failed to identify factors for groups that had a small number of events. Second, we did not perform an analysis based on levels of disability. Among the participants with newly occurring functional disability, 42.4% were categorized as low level and 57.6% as higher levels. In this study, the percentage of participants with higher-level functional disability was higher than that found in precedence reports. One possible reason for this difference is that the participants in our study were all 70 years or older, not 65 or older. Third, because we proceeded from the statistical hypothesis that physical, mental, and social factors at the baseline would be predictive of future functional disability in elderly people living independently in a community, we did not collect data on all episodes of the onset of functional disability, and so we did not analyze them for time-varying covariates. So there could have be an underestimation regarding the onset of functional disability. In addition, no significant association was detected between the genders with respect to functional disability in any of the household groups; thus, no additional analysis according to gender was performed. It is necessary to conduct an analysis that takes into account both the care level needed and gender. In addition, although the participants of this study were all aged 70 or older, Takeda [38] reported that the percentage of disability increases drastically in the over-75 age-group and that deterioration after certification of long-term care is dominant in the over-80 age-group. Thus, using a large scale cohort including elderly people aged above 85, we need to analyzed the age stratified group in each household group. Health service authorities have focused on elderly people living alone or in couple-only households as a result of smaller family sizes and decreased family functions. However, such authorities have little information about elderly individuals living only with their children. These children-only households showed the highest rate of functional disability in our results. From the perspectives of the elderly, the reasons for this could be advanced age, loss of the spouse, and deteriorating health; from the perspectives of the children, the reasons may be related to economic status or mental and physical status. We did not collect data related to the children’s characteristics, age, sex, marital status, or health condition. Future studies need to clarify such details with respect to middle-aged and older adults living with their elderly parents. Despite these limitations, this is the first study in Japan to provide new information about the risk factors of functional disability according to household composition. It was found that there was vulnerability both among elderly people living alone and among those living in a household only with their children.

## Conclusions

We examined how risk factors of functional disability among elderly people differed according to four types of household composition. Those living in households only with their children demonstrated a significantly higher risk for functional disability than the three-generation household group (HR, 1.61; 95% CI, 1.08–2.40). The risk factors for functional disability differed according to household composition. Community-based nursing needs a means of assessment that takes household composition into consideration to appropriately identify the risk factors related to functional disability among community-dwelling elderly people.

## Endnotes

The MFS comprises three subscales: mobility (items a–f); strength (h–k); and balance (l–o).

^a^I can walk up to and down from the second floor.

^b^I can walk up to the second floor without getting out of breath.

^c^I can jump up in the air so that both feet are clearly off the floor at the same time.

^d^I can run for 20 paces.

^e^I can pass another person who is walking ahead of me.

^f^I can keep walking for over 30 minutes.

^h^I can carry something weighing 10 pounds (e.g., a 1-gallon milk bottle).

^i^I can lift something weighing 20 pounds (e.g., two 1-gallon milk bottles).

^j^I can pick up a fallen bicycle.

^k^I can open a screw-type bottle cap.

^l^I can touch the floor with my fingertips while standing with extended knees.

^m^I can put on a sock, slacks, or a skirt while standing with no support.

^n^I can stand up from a chair without using my hands.

^o^I can stand on my toes without any support.

## Competing interests

The authors declare that they have no competing interests.

## Authors’ contributions

ES collected data, performed all statistical analyses, and had primary responsibility for writing this paper. SU collected data and contributed to revising the paper. NY planned the study and contributed to revising the paper. SY collected data, coordinated with interviewers, and contributed to revising the paper. SY planned the study, supervised the data analysis, and aided ES in interpreting the clinical significance of the results. SY also participated in writing this paper. All the authors read and approved the final manuscript.

## Pre-publication history

The pre-publication history for this paper can be accessed here:

http://www.biomedcentral.com/1471-2318/14/93/prepub
